# Validation study of risk-reduction activities after personalized breast cancer education tool in the WISDOM study

**DOI:** 10.1038/s41523-024-00681-z

**Published:** 2024-10-14

**Authors:** Tianyi Wang, Mandy Che, Yash S. Huilgol, Holly Keane, Deborah Goodman, Rashna Soonavala, Elissa Ozanne, Yiwey Shieh, Jeffrey K. Belkora, Allison Stover Fiscalini, Athena Breast Health Network Investigators and Advocate Partners, Laura Esserman, Laura van ‘t Veer, Robert Hiatt, Jeff Tice, Elad Ziv, Amie Blanco, Barry Tong, Katherine Ross, Allison Fiscalini, Maren Scheuner-Purcell, Kimberly Badal, Kim Rhoads, Celia Kaplan, Christina Yau, Rashna Soonavala, Katherine Leggat-Barr, Tomiyuri Lewis, Patricia Choy, Steffanie Goodman, Leah Sabacan, Kenneth Wimmer, Kelly Adduci, Stephanie Flores, Roxanna Firouzian, Irene Acerbi, Arash Naeim, Neil Wenger, Carlie Thompson, Antonia Petruse, Annette Stanton, Alexander Borowsky, Skye Stewart, Lydia Howell, Hoda Anton-Culver, Hannah Lui Park, Deborah Goodman, Lisa Madlensky, Andrea LaCroix, Barbara Parker, Tracy Layton, Michael Hogarth, Sheri Hartman, Diana DeRosa, John Pierce, Andrea Kaster, Jan Wernisch, Olufunmilayo Olopade, Rachael Lancaster, James Esserman, Martin Eklund, Yiwey Shieh, Karen Sepucha, Vivian Lee, Diane Heditsian, Susie Brain, Dolores Morehead, Laura J. Esserman

**Affiliations:** 1 UC San Francisco Department of Surgery, San Francisco, CA USA; 2 University of Michigan Medical School, Ann Arbor, MI USA; 3 Rush University Medical College, Chicago, IL USA; 4 UC San Francisco School of Medicine, San Francisco, CA USA; 5 Peter MacCallum Cancer Centre, Melbourne, VIC Australia; 6 UC Irvine Department of Epidemiology, Irvine, CA USA; 7 University of California—Los Angeles School of Medicine, Los Angeles, CA USA; 8 University of Utah School of Medicine Department of Population Health Sciences, Salt Lake City, UT USA; 9 Weill Cornell Medicine Department of Population Health Sciences, New York, NY USA; 10 University of California, San Francisco, CA USA; 11 Kannact, Albany, NY USA; 12 Wildflower Health, San Francisco, CA USA; 13 Exai Bio, Palo Alto, CA USA; 14 University of California, Los Angeles, CA USA; 15 University of California, Davis, CA USA; 16 University of California, Irvine, CA USA; 17 University of California, San Diego, CA USA; 18 Sanford Health, Sioux Falls, SD USA; 19 University of Chicago, Chicago, IL USA; 20 University of Alabama Birmingham, Birmingham, AL USA; 21 Diagnostic Center of Miami, Miami, FL USA; 22 Karolinska Institutet, Solna, Sweden; 23 Weill Cornell Medicine, New York, NY USA; 24 Mass General, Boston, MA USA; 25 WISDOM Advocate, San Francisco, CA USA

**Keywords:** Breast cancer, Risk factors

## Abstract

Breast cancer risk reduction strategies have been well-validated, but barriers remain for high-risk individuals to adopt them. We performed a study among participants with high risk of breast cancer to validate whether a virtual breast health decision tool impacted a participant’s willingness to start risk-reducing activities, identify barriers to adopting these strategies, and understand if it affects breast cancer anxiety. The study sample was 318 participants in the personalized (investigational) arm of the Women Informed to Screen Depending on Measures of risk (WISDOM) clinical trial. After reviewing the tool, these participants completed a feedback survey. We demonstrated that 15 (4.7%) women were taking endocrine risk reduction, 123 (38.7%) were reducing alcohol intake, and 199 (62.6%) were exercising. In the three-month follow-up survey of 109 respondents, only 8 of 61 (13.1%) women who considered endocrine risk reduction pursued it. In contrast, 11 of 16 (68%) participants who considered alcohol reduction pursued the activity, and 14 of 24 (58%) women who considered exercise followed through. Participants listed fear of side effects as the most common barrier to endocrine risk reduction. We also present further steps to be taken to improve the effectiveness of the Breast Health Decisions tool.

## Introduction

Validated strategies to reduce breast cancer risk include lifestyle changes (reducing alcohol intake, increasing exercise, losing weight), endocrine risk reduction medications (selective estrogen receptor modulators and aromatase inhibitors), and avoidance of combined hormone replacement after menopause^1–16^. While lifestyle modifications are recommended for all women, the United States Preventative Task Force encourages endocrine risk reduction in high-risk women over age 35^1–5^. Despite having proven prevention strategies, those who stand to benefit often lack appropriate counseling. Adherence uptake of breast cancer endocrine risk reduction in the United States remains low, partly due to lack of education, health literacy, and concerns about treatment^5–9^.

Educational risk assessment tools can provide personalized breast cancer risk knowledge^10^. In the clinical setting, such tools improve provider-patient communication when deciding on risk-reducing interventions^3^. Previously, members of our team developed the Breast Health Decisions (BHD) tool, which educates participants with elevated breast cancer risk using accessible natural frequency language and visuals of absolute risk^11,12^. The tool is available to eligible participants of the Women Informed to Screen Depending on Measures of risk (WISDOM) Study. Its purpose is to encourage risk-reducing behaviors and evaluate one of the WISDOM Study’s secondary endpoints: whether understanding personalized risk results in the uptake of breast cancer prevention strategies.

Here, we describe the results of the validation study of the BHD tool in participants with the highest 2.5% breast cancer risk based on Breast Cancer Surveillance Consortium (BCSC) scores in the WISDOM Study. The study builds upon our prior pilot study and evaluates the adoption of risk-reducing strategies over a three-month period after counseling with the BHD tool. We sought to answer the following questions:

1. Is the use of the BHD tool in women with high breast cancer risk associated with consideration and initiation of risk-reducing activities?
2. What are the barriers to risk-reducing activities among high-risk women after the use of the BHD tool?
3. To what extent does the risk assessment tool affect breast cancer anxiety in high-risk women?

## Results

### Descriptive statistics

The 318 study participants had an average age of 57.8 with a standard deviation of 9.5. The majority of participants were white (283, 89%), had a college degree or higher (244, 76.7%), and had a body mass index within the range of 18.5 to 24.9 (179, 56.3%) (Table 1). Within the cohort, 221 participants were in the high-risk screening category, while 97 fell into the highest-risk category. High-risk participants had an average BCSC score of 5.10, and highest-risk participants had an average score of 7.62. Among the 318 participants, 109 responded to the three-month follow-up survey, with 72 categorized as high-risk and 37 as highest risk.

**Table 1:** Baseline characteristics of study participants

		High risk *N* = 221 (%)	Highest risk *N* = 97 (%)	Total participants *N* = 318 (%)
Age	40–49	64 (29%)	7 (7.2%)	71 (22.3%)
	50–59	72 (32.6%)	34 (35%)	106 (33.3%)
	60–69	53 (24%)	49 (50.5%)	102 (32.1%)
	70–79	32 (14.4%)	7 (7.3%)	39 (12.3%)
BMI	<18.5	2 (0.9%)	4 (4.1%)	6 (1.9%)
	18.5–24.9	120 (54.3%)	59 (60.8%)	179 (56.3%)
	25–29.9	58 (26.2%)	18(18.6%)	76 (23.9%)
	>30	41 (18.6%)	16 (16.5%)	57 (17.9%)
Race/ethnicity	White	196 (88.7%)	87 (89.7%)	283 (89%)
	Hispanic	5 (2.3%)	1 (1.0%)	6 (1.9%)
	Black or African American	5 (2.3%)	0	5 (1.6%)
	Asian	2 (0.9%)	3 (3.1%)	5 (1.6%)
	Native Hawaiian or Other Pacific Islander	1 (1.3%)	0	1 (0.31%)
	Two or more races	10 (4.5%)	3 (3.1%)	13 (4.1%)
	Some other race	1 (0.5%)	2 (2.1%)	3 (0.94%)
	No response	0	1 (1.0%)	1 (0.31%)
	Prefer not to answer	1 (0.5%)	0	1 (0.3%)
Education	High school	7 (3.2%)	2 (2.1%)	9 (2.8%)
	College or technical school	41 (18.6%)	23 (23.7%)	64 (20.1%)
	College graduate or more	173 (78.2%)	71 (73.2%)	244 (76.7%)
	No response	0	1 (1%)	1 (0.4%)

Age, BMI, race/ethnicity, education of participants, and further subset for high- and highest-risk participants. Percentages (%) were calculated in each column according to the column’s total *N*.

### Aim 1: Risk assessment tool and risk-reducing activities

**Fig. 1:**
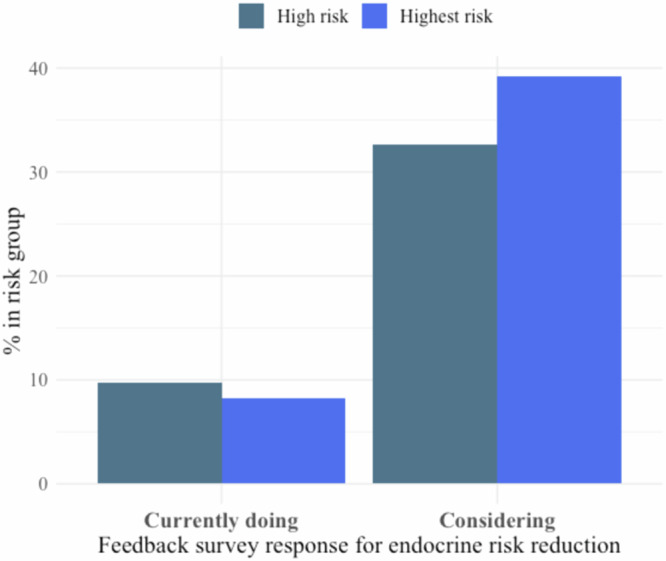
Feedback survey of individuals using and considering endocrine risk reduction. Bar graph of individuals taking endocrine risk reduction (left) and those considering endocrine risk reduction, as answered on the feedback survey. Data were subset into high and highest-risk participants and presented as percentages out of total *N* in the risk group.

In the immediate feedback survey, most participants (98.4%) found the tool effective in understanding their breast cancer risk (Supplementary Table 1). Of the 318 participants, 15 (4.7%) were currently taking endocrine risk reduction, 123 (38.7%) were reducing alcohol intake, and 199 (62.6%) were engaging in exercise out of the total 318 participants. Of the 221 high-risk participants, 7 (3.2%) were taking endocrine risk reduction medication, 74 (33.5%) were decreasing alcohol, and 133 (60.2%) were increasing exercise. Of the 97 highest-risk participants, 8 (8.2%) were using endocrine risk reduction, 49 (50.5%) were decreasing alcohol use, and 66 (68%) were increasing exercise. Of note, Pearson’s chi-squared test found highest-risk participants had a significantly higher uptake of alcohol reduction than high-risk women. After using the BHD tool, 110 participants in the immediate feedback survey (34.6%) said they were considering taking endocrine risk reduction, 47 (14.8%) contemplated reducing alcohol consumption, and 98 (30.8%) thought about increasing exercise (Table 2, Fig. 1).

**Table 2:** Immediate feedback and follow-up surveys: use and considerations of breast cancer risk-reducing activities

Immediate feedback						
	Screening category			Pearson’s Chi-squared test (high vs. highest-risk participants)		
	High (*N* = 221)	Highest (*N* = 97)	Total (*N* = 318)	Chi-square	*p* value	*df*
Current risk-reducing activities						
Use endocrine risk reduction	7 (3.2%)	8 (8.2%)	15 (4.7%)	2.8	0.09	1
Decrease alcohol	74 (33.5%)	49 (50.5%)^**≠**^	123 (38.7%)	7.5	0.006	1
Increase exercise	133 (60.2%)	66 (68%)	199 (62.6%)	1.5	0.84	1
Lose weight	82 (37.1%)	45 (46.4%)	127 (39.9%)	N/A	N/A	N/A
Other	14 (6.3%)	12 (12.4%)	26 (8.2%)	N/A	N/A	N/A
Nothing	52 (23.5%)	13 (13.4%)	65 (20.4%)	N/A	N/A	N/A
Risk-reducing activities under consideration						
Use endocrine risk reduction	72 (32.6%)	38 (39.2%)	110 (34.6%)	1.02	0.31	1
Decrease alcohol	33 (14.9%)	14 (14.4%)	47 (14.8%)	9.5 × 10^−30^	1	1
Increase exercise	76 (34.4%)	22 (22.7%)	98 (30.8%)	3.8	0.051	1
Lose weight	65 (29.4%)	17 (17.5%)	82 (25.8%)	N/A	N/A	N/A
Other	14 (6.3%)	3 (3.1%)	17 (5.3%)	N/A	N/A	N/A
Nothing	42 (19%)	22 (22.7%)	64 (20.1%)	N/A	N/A	N/A

Three-month follow-up						
Current risk-reducing activities	Screening category			Pearson’s Chi-squared test (high vs. highest-risk participants)		
	High (*N* = 72)	Highest (*N* = 37)	Total (*N* = 109)	Chi-square	*p* value	*df*
Use endocrine risk reduction	7 (9.7%)	5 (13.5%)	12 (11%)	0.076	0.78	1
Decrease alcohol	26 (36.1%)	16 (43.2%)	42 (38.5%)	0.27	0.6	1
Increase exercise	34 (47.2%)	19 (51.4%)	53 (48.6%)	0.042	0.84	1
Improve diet	47 (65.3%)	26 (70.3%)	73 (67%)	N/A	N/A	N/A
Would like support services	30 (41.7%)	17 (45.9%)	47 (43.1%)	N/A	N/A	N/A

Current risk-reducing activities of participants as answered on the feedback survey, and risk reducing activities under consideration. Participants could select more than one activity, so percentages do not add up to 100. Percentages (%) calculated in each column according to column’s total *N*. High-risk participants were defined as yearly mammography screening assignments on the WISDOM Study. Highest risk participants were defined as every 6-month screening (alternating mammography and MRI) on the WISDOM Study. For statistical analysis, Pearson’s chi-squared test with Yates’ continuity correction was performed between high and highest-risk groups, with *p* values listed in the last column and ≠ designating statistical significance between the two groups.

Of the 109 participants who completed the follow-up survey, 12 (11%) were using endocrine risk reduction medications, 42 (38.5%) had decreased alcohol intake, and 53 (48.6%) had increased exercise. Of the 72 high-risk participants, 7 (9.7%) were using endocrine risk reduction, 26 (36.1%) were decreasing alcohol, and 34 (47.2%) were increasing exercise. Of the 37 highest-risk participants, 5 (13.5%) were using endocrine risk reduction, 16 (43.2%) had decreased alcohol consumption, and 19 (51.4%) were exercising. Pearson’s chi-squared test found no significant difference between high and highest-risk groups (Table 2).

Of the 61 participants who considered endocrine risk reduction in the immediate feedback survey, 8 (13.1%) started it three months later. Among the 48 who did not consider it, 4 (8.4%) began taking it three months later (Supplementary Table 3). 11 of 16 participants (68.7%) who considered reducing alcohol intake initiated it three months later, compared to 31 of 93 participants (33.3%) who did not consider it (Supplementary Table 4). Lastly, 14 of 24 participants (58.3%) who considered increasing exercise did so three months later, while 39 of 85 women (45.9%) who did not consider it began doing so (Supplementary Table 5).

### Aim 2: Barriers to provider discussion and risk-reducing activities

Among the 109 women who completed their three-month follow-up survey, 80 (73.3%) engaged in breast cancer risk discussions with their healthcare providers (Table 3). Among these 80 participants, 19 (23.8%) were advised to pursue endocrine risk reduction, 15 (18.8%) were recommended to reduce alcohol intake, and 22 (27.5%) were encouraged to increase exercise (Table 3). Of 72 high-risk women who completed the follow-up survey, 50 discussed risk with their providers. Among the 50 participants, 11 (22%) received recommendations for endocrine risk reduction, 9 (18%) for alcohol reduction, and 11 (22%) for exercise increase. Of the 37 participants in the highest-risk group, 30 discussed their risk with providers, with 8 (26.7%) being advised to take endocrine risk reduction medication, 6 (20%) to reduce alcohol intake, and 11 (36.7%) to increase exercise (Table 3). 15 of 29 participants (51.7%) did not talk about risk with their providers, and the reasons cited for not having the discussions were pending or unscheduled appointments or lack of topic initiation during their appointment (Supplementary Table 6).

**Table 3:** Healthcare risk-reducing recommendation for moderate and high-risk women

	High risk *N* = 72 (%)	Highest risk *N* = 37 (%)	Total *N* = 109 (%)
Discussed risk with provider	50 (69.4%)	30 (81.1%)	80 (73.3%)
Risk-reduction strategy recommended by healthcare provider			
Use endocrine risk reduction	11 (15.3%)	8 (21.6%)	19 (17.4%)
Decrease alcohol	9 (12.5%)	6 (16.2%)	15 (13.8%)
Increase exercise	11 (15.3%)	11(29.7%)	22 (20.2%)
Lose weight	11 (15.3%)	4 (10.8%)	15 (13.8%)
Other	8 (11.1%)	4 (10.8%)	12 (11%)
Nothing at this time	18 (25%)	8 (21.6%)	26 (23.9%)

Includes risk reducing recommendations (use medication, decrease alcohol, increase exercise, lose weight, other, nothing at this time) by healthcare providers. Pearson’s chi-squared test with Yates’ continuity correction was performed between high- and highest-risk groups.

Key barriers to initiating endocrine risk reduction medication discussion included “other” (44 participants, 45.4%) and “fear of side effects” (36 participants, 37.1%) (Supplementary Table 6). Within the “other” category, most women mentioned that the provider did not recommend the medication. Most women not initiating a lifestyle risk-reducing strategy reported they were already performing these activities (Supplementary Table 6).

### Aim 3: Emotional well-being after use of the risk assessment tool

Among the total 318 participants who used the tool, 139 (43.7%) participants reported reduced anxiety about their breast cancer risk, while 122 (38.4%) women remained neutral about the tool’s impact, and 52 (16.3%) disagreed with its effectiveness in alleviating anxiety (Supplementary Table 1). Out of the 221 high-risk participants, 95 (43%) reported a benefit of lower anxiety, 83 (37.6%) were neutral, and 38 (17.1%) did not perceive any benefit. Among the 97 highest-risk participants, 44 (45.3%) found anxiety relief, and 39 (40.2%) expressed neutrality.

Among the 109 participants who completed the three-month follow-up survey, 10 (9.2%) often thought about their chances of developing breast cancer, 70 (64.2%) sometimes thought about their chances, and 28 (25.7%) did not think about their chances at all. In terms of worries about developing breast cancer, none of the participants worried almost all the time, 6 (5.5%) often worried, 53 (48.6%) sometimes worried, and 49 (45%) did not worry at all (Supplementary Table 2).

## Discussion

Our group developed and tested a comprehensive decision support tool, BHD, to support education and risk-reducing choices for women at high risk for breast cancer. Most participants found the tool aided their understanding of breast cancer risk, suggesting efficacy in natural language frequencies to express and visualize risk. In addition, the BHD tool facilitated the desire to begin endocrine risk reduction in many women at high risk for breast cancer (110, 34.6%). The tool also facilitated the consideration of increasing exercise (123, 38.7%). While a lower proportion considered alcohol reduction (47, 14.8%) after using the tool, this may be because many participants already reduced their alcohol intake and were less inclined to reduce it further (123, 38.7%) (Table 2).

Even among participants who considered endocrine risk reduction, uptake of risk-reducing medication remained low at three months. Only 8.4% of women who considered endocrine risk reduction pursued it three months later, in contrast to the proportion of individuals who considered and then pursued lifestyle changes (30–50%) (Supplementary Tables 3–5). Our study suggests this discrepancy was predominantly due to fear of medication side effects, which was the most frequently identified barrier to endocrine risk reduction.

In addition, many women who did not pursue endocrine risk reduction reported that they either did not have a follow-up visit with their healthcare provider, or the topic was not brought up. Three months may have been insufficient time for many women to schedule and attend healthcare appointments. Also, lack of time in healthcare appointments may have prevented the initiation of the topic. Furthermore, of the 80 high-risk women who discussed their breast cancer risk with providers, only 19 (23.8%) were advised to pursue endocrine risk reduction, 15 (18.8%) were recommended alcohol reduction, and 22 (27.5%) were encouraged to increase exercise. This finding raises the question of whether healthcare providers could benefit from further support in discussing risk-reducing strategies. Past studies also suggest barriers to endocrine risk reduction medication uptake may be at the provider level in the clinic^7,8,13^. When assessing risk, most providers never calculate Gail scores (76%). While many discuss risk in high-risk women (58%) and tailor screening based on risk (53%), fewer providers discuss endocrine risk reduction (13%)^13^. Challenges faced by providers include lack of confidence in risk assessment, identification of suitable candidates, insufficient knowledge of risk-reducing medications, more urgent issues, and lack of time^7,8,13^. Therefore, despite current clinical guidelines, not all high-risk women may be targeted for endocrine risk reduction during their healthcare provider appointments.

**Fig. 2:**
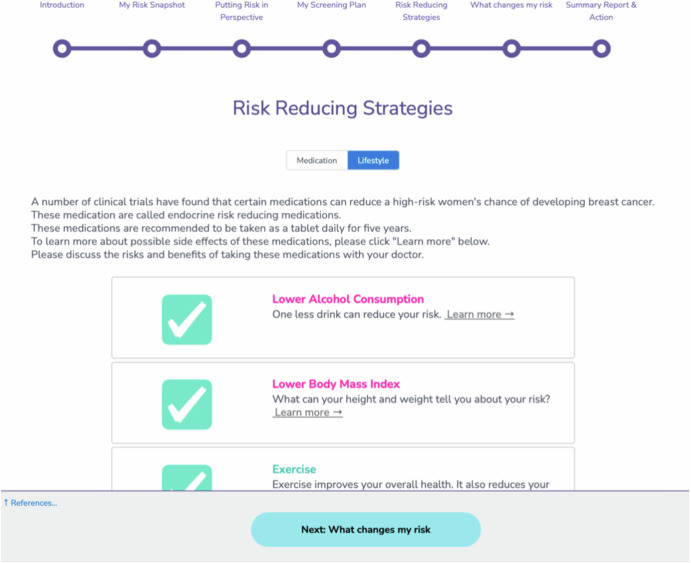
Breast Health Decisions tool page examples.

Since healthcare providers are often women’s most trusted source of health information, the application of breast cancer risk assessment tools in the clinical setting will require education of and collaboration with the providers directly involved in patient care^9,14,15^. This proposal would emulate the adoption of heart disease risk assessment by primary care physicians, which reduced cardiac-related mortality risk by 50% over the past several decades^16,17^. Alternatively, providing women with virtual prevention clinics could improve Fig. 2 medication uptake.

**Fig. 3:**
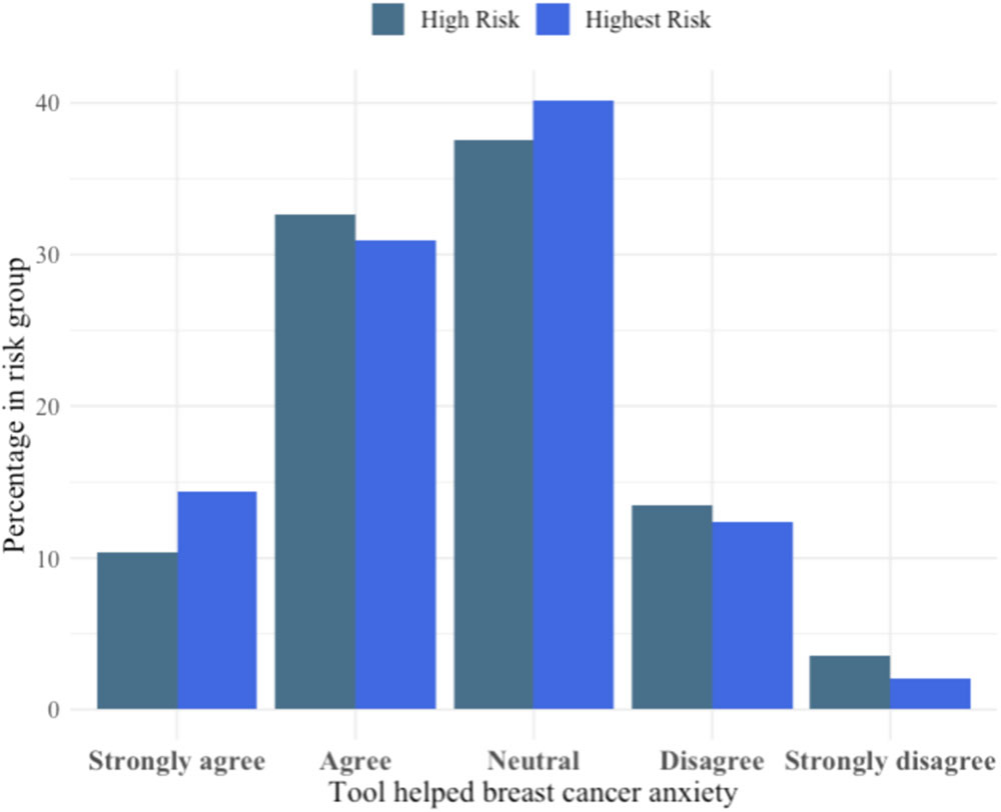
Feedback survey breast cancer anxiety. Bar graph about whether the Breast Health Decisions tool eased breast cancer anxiety, as answered on the feedback survey. Responses were presented on a Likert Scale (strongly agree, agree, neutral, disagree, strongly disagree). Data were subset into high and highest-risk participants and presented as percentages out of total *N* in the risk group.

**Fig. 4:**
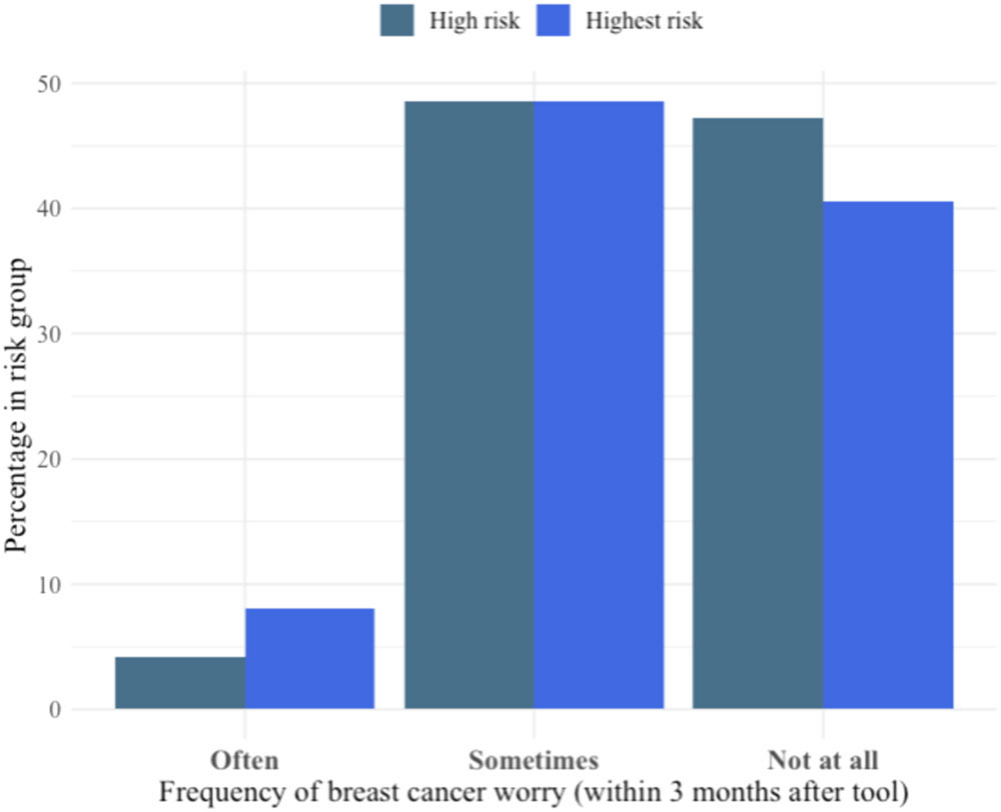
Three-month follow-up survey, frequency of breast cancer worry. Bar graph of the frequency of breast cancer worry, as answered on the 3-month follow-up survey. Responses were obtained through Likert Scale (often, sometimes, not at all). Data were subset into high- and highest-risk participants and presented as percentages out of the total *N* in the risk group.

Anxiety and worry negatively impact decision-making and are especially prevalent in women with a family history of breast cancer, baseline anxiety, negative illness perceptions, and genetic testing^18–23^. Prior studies demonstrate that providing women with breast cancer risk estimates has minimal negative effects on anxiety^24,25^. However, it is unclear if actionable risk reduction strategies from educational tools like the BHD tool can have a positive effect^21,24,25^. In this preliminary investigation of breast cancer risk anxiety and worry after use of an educational tool, majority of women reported no negative effect on their emotional state (Figs. 3–4, Supplementary Table 2). These findings suggest that risk knowledge is not associated with negative emotions and may even alleviate breast cancer anxiety. It is possible that providing risk reduction strategies empowers women, thus positively contributing to emotional well-being.

Our study has several limitations. First, the COVID-19 pandemic began during our data collection process, so results may be confounded by the lockdown and closure of gyms and recreational centers, making it difficult to attend healthcare appointments and maintain lifestyle routines^26–29^. Second, the study was based on survey data and self-report, which poses possible reporting bias. Survey questions also did not include questions asking participants to quantify their risk-reducing activity. Third, our follow-up survey sample response rate was 35%, which raises the possibility of response or attrition bias. In addition, we only assessed follow-up at the 3-month mark. Therefore, we could not ascertain participants who began a behavior change and discontinued it before three months, nor participants who discontinued the behavior after three months.

We also acknowledge additional factors limit generalizability. Our study sample is a small minority of WISDOM Study participants in the personalized arm, and may share characteristics not reflective of the general population. Furthermore, our participants were predominantly white and highly educated, with no Blacks in the highest-risk group. And lastly, by design, we did not include participants who were high risk by virtue of pathogenic genetic variants.

To directly involve and partner with primary care clinicians, we are working with primary care groups to optimally share risk assessment information and determine if a virtual prevention program can support primary care providers in educating patients. To better quantify lifestyle changes, we hope future studies can include survey questions quantifying alcohol intake and minutes exercised per week and explore the use of health app data. We have also modified the BHD tool to educate women about the potential to take a lower dose of tamoxifen, as it has been shown to be much more tolerable and equally effective in the setting of DCIS. Furthermore, we have increased the diversity of WISDOM Study participants. In future studies, we hope to have increased study diversity with multiple follow up time points and a longer study duration.

## Methods

### The WISDOM study

The WISDOM Study, approved by the University of California, San Francisco (UCSF) Institutional Review Board (approval #15-18234), is an adaptive, randomized clinical trial comparing the comprehensive risk-based (personalized) approach to annual breast cancer screening. The trial aims to determine whether screening based on personalized risk is as safe or less morbid, preferred by women, and will facilitate prevention for those most likely to benefit^12^. To evaluate whether understanding personalized risk facilitates prevention, the study investigators created a risk assessment tool for eligible participants. Participants of the WISDOM Study provided digital written informed consent, including the option to complete additional surveys. Registered on ClinicalTrials.gov as NCT02620852, the study is conducted online through a secure Salesforce-based platform.

### Breast health decision tool and modifications

Previously, our team reported the development and pilot of the BHD tool.^33^ This tool is accessible via participants’ WISDOM Study portals. It encompasses five primary pages—*my risk snapshot, my risk report, putting risk in perspective, risk-reducing strategies*, and *exploring what changes my risk*—each displaying an increasingly nuanced perspective of participants’ breast cancer risk and risk mitigation strategies^11^. The primary pages provide extra hyperlinked information to peer-reviewed articles and a personalized risk report in PDF format available on the summary page. Following the initial pilot involving 17 participants, minor visual and software adjustments were made, including updating references incorporating new data on endocrine risk reduction, the visual interface, and editing the “Exploring Factors Affecting My Risk” page to visually showcase each strategy’s positive risk mitigation effects according to each participants’ unique risk and WISDOM survey responses.

### Participant selection

The eligible participants for the WISDOM Study are women aged 40 to 74 residing in the United States with no prior breast cancer diagnoses. The validation study comprised 318 participants from the personalized arm of the WISDOM Study, classified as elevated risk (top 2.5%) BCSC scores by age and without pathogenic variants in breast cancer susceptibility genes (*BRCA1, BRCA2, TP53, PTEN, STK11, CDH1, ATM, PALB2, CHEK2*). Women in the high-risk category had a 5-year risk greater than or equal to 6% if they were aged 65 or older or had a biopsy with atypia and first-degree family history without chemoprevention. Women in the highest-risk category had a 5-year risk greater than or equal to 6% if they were aged 40–64 years old or had a history of chest wall radiation before age 35 (Table 1). The former was recommended annual mammograms, while the latter were advised annual mammograms along with annual MRI screenings.

### Study procedure

Data collection spanned from February 2019 to April 2022. Participants engaged with the tool at their own pace once accessible on their portal. According to the WISDOM Study protocol, a virtual breast health specialist annually contacted high risk participants via email or phone, facilitating a zoom meeting to navigate through the tool. Participants not responding or declining could still independently access the tool.

Following initial tool use, an immediate feedback survey appeared on the last page. Completing this survey triggered a follow-up survey in their portal three months later. Our total study sample was 333 participants responding to the feedback survey. We excluded 2 participants with “stop screening” or “start screening at age 50” recommendations and 13 who completed their survey after being designated low-risk (re-calibration of participant risk is performed yearly). Of the 318 participants included in the immediate feedback survey, 109 responded to the three-month follow-up survey.

### Immediate feedback survey

The immediate feedback survey comprised five questions addressing participant insights into their personal breast cancer risk following tool usage, concerns about breast cancer, and risk-reducing activities. Questions 1, 2, and 3 were rated on a Likert scale (*strongly agree, agree, neutral, disagree, strongly disagree),* while questions 4 and 5 featured checkboxes with options: *taking medication that reduces my risk, decreasing alcohol intake, increasing exercise, losing weight, other (fill in blank), nothing at this time*. A summary of the five questions is below (see supplementary material for a full survey):

1. Understanding my chance of breast cancer after using a risk assessment tool
2. Worry and anxiety about breast cancer risk after using risk assessment tool
3. Desire to lower breast cancer risk
4. Current risk-reducing activities
5. Considering the following risk-reducing activities after using a risk assessment tool

### Three-month follow-up survey

The three-month follow-up survey encompassed 9 questions concerning worry about breast cancer, healthcare provider discussions, risk-reducing activities, and barriers. Questions concerning worry about breast cancer used a Likert scale (*not at all, sometimes, often, almost all of the time)*. Questions regarding the desire to lower breast cancer risk and the efficacy of the BHD tool in influencing decisions employed a Likert scale (*strongly agree, agree, neutral, disagree, strongly disagree)*. Questions regarding risk-reducing activities had checkboxes (*risk-reducing medication, decreasing alcohol intake, increasing exercise, losing weight, other, nothing at this time)*. Questions regarding barriers were checkbox items with the following selections: *I do not need to reduce my chance, I am already taking steps to reduce my chance, personal motivation, financial barriers, access to a health provider, time limitations, other* (see supplementary material for a full survey).

### Data analysis

Data analyses were performed using R Studio version 1.0.153. Pearson’s chi-squared test was performed between high and highest-risk group data. All 318 participants from the immediate feedback survey were included in tables and analyses specific to that survey. Tables and figures comparing feedback and follow-up survey results were confined to the 109 participants who responded to the follow-up survey.

Study coordinator MC downloaded an immediate feedback survey, 3-month follow-up survey data, and participant demographics information from the Salesforce platform. Study coordinator TW compiled the demographics and survey information into tables and figures and performed analytics using R.

### Ethics

The study complied with all relevant ethical regulations and in accordance with the principles of the Declaration of Helsinki.

### Reporting summary

Further information on research design is available in the Nature Research Reporting Summary linked to this article.

## Supplementary information

Supplemental material

Reporting summary

## Data Availability

The datasets used and analyzed for the current study are available on Open Science Framework and can be accessed from the following link: 10.17605/OSF.IO/YJ782.

## Code availability

The underlying code for this study is available on Zenodo and can be accessed through the following link: 10.5281/zenodo.8374402.
