# Supplementary material for: Validation study of risk-reduction activities after personalized breast cancer education tool in the WISDOM study
Source: NPJ Breast Cancer. 2024 Oct 14;10:90. doi: 10.1038/s41523-024-00681-z (PMC11471852; doi:10.1038/s41523-024-00681-z)
Supplement: Supplementary file 1 — Supplemental material [file 41523_2024_681_MOESM1_ESM.pdf]

Supplementary Table 1: Tool Efficacy in Understanding Risk and Easing Anxiety

Tool helped me understand my breast cancer risk	Moderate risk (N = 221)	High risk (N = 97)	Total (N = 318)
Strongly agree	98 (44.3%)	49 (50.5%)	147 (46.2%)
Agree	119 (53.8%)	47 (48.5%)	166 (52.2%)
Neutral	0	0	0
Disagree	0	1 (1%)	1 (0.3%)
Strongly disagree	3 (1.4%)	0	3 (1%)
No response	1 (0.5%)	0	1 (0.3%)
BHD Tool eased worries and anxiety about breast cancer risk *Note: data is reflected in figure 4*			
Strongly agree	23 (10.4%)	14 (14.4%)	37 (11.6%)
Agree	72 (32.6%)	30 (30.9%)	102 (32.1%)
Neutral	83 (37.6%)	39 (40.2%)	122 (38.4%)
Disagree	30 (13.5%)	12 (12.4%)	42 (13.2%)
Strongly disagree	8 (3.6%)	2 (2.1%)	10 (3.1%)
No response	5 (2.3%)	0	5 (1.6%)

Supplementary Table 2: Thoughts and Worries about Breast Cancer (3-month follow up)

	Screening category		
	Moderate risk (N = 72)	High risk (N = 37)	Total (N = 109)
Thought about my chances of developing breast cancer			
Often	6 (8.3%)	4 (10.8%)	10 (9.2%)
Sometimes	45 (62.5%)	25 (67.6%)	70 (64.2%)
Not at all	21 (29.2%)	7 (18.9%)	28 (25.7%)
No response	0 (%)	1 (2.7%)	1 (0.9%)
Worried about my chances of developing breast cancer **Note: data is reflected in Figure 5*			
Almost all of the time	0	0	0
Often	3 (4.2%)	3 (8.1%)	6 (5.5%)
Sometimes	35 (48.6%)	18 (48.6%)	53 (48.6%)
Not at all	34 (47.2%)	15 (40.5%)	49 (45%)
No response	0	1 (2.7%)	1 (0.9%)

Supplementary Table 3: Feedback vs. Follow Up Survey in Endocrine Risk Reduction

		3 month follow up survey: taking endocrine risk reduction?		Total
		No	Yes	
Feedback survey: considering endocrine risk reduction?	No	43 (89.6%)	4 (8.4%)	48
	Yes	53 (86.9%)	8 (13.1%)	61

Supplementary Table 4: Feedback vs. Follow Up Survey in Alcohol Intake

		3 month follow up survey: decreasing alcohol intake?		Total
		No	Yes	
Feedback survey: considering decreasing alcohol?	No	61 (65.6%)	31 (33.3%)	93
	Yes	5 (31.3%)	11 (68.7%)	16

Supplementary Table 5: Feedback vs. Follow Up Survey in Exercise

		3 month follow up survey: increasing exercise?		Total
		No	Yes	
Feedback survey: considering increasing exercise?	No	45 (52.9%)	39 (45.9%)	85
	Yes	10 (41.7%)	14 (58.3%)	24

Supplementary Table 6: Barriers to Risk Reducing Activities

		Number of participants	Percentage out of total (%)
Reason for not discussing with provider			*N = 29*
	No need	2	6.9
	Motivation	3	10.3
	Finance	0	N/A
	Access	7	24.1
	Time	4	13.8
	Other<br>- Not brought up in appointment (5)<br>- Appointment has not occurred (8)<br>- Dealing with another medical condition (1)<br>- Do not have a physician (1)	15	51.7
Reason for not using endocrine risk reduction			*N = 97*
	No need	15	15.5
	Motivation	5	5.2
	Finance	1	1.0
	Time	1	1.0
	Access	7	7.2
	Fear of side effects	36	37.1
	Other	44	45.4

	- Appointment has not occurred (3)<br>- recommendations (2)<br>- Trying something else (3)<br>- Not recommended by provider (16)<br>- Undecided (5)<br>- I fertility treatment (1)<br>- Lack of knowledge (2)<br>- Does not want to (3)<br>- Tried it before (2)<br>- Dealing with another medical condition (2)		
Reason for not reducing alcohol			*N = 67*
	No need	0	N/A
	Already do it	53	79.1
	Motivation	8	11.9
	Other	5	7.5
Reason for not exercising more			*N = 56*
	No need	1	1.8
	Already do it	36	64.3
	Motivation	10	17.9
	Time	4	7.1
	Access to personal trainer	1	1.8

WISDOM Study Breast Health Decisions Tool Surveys

<u>Feedback Survey (immediately after using tool)</u>

1. **I have a better understanding of my chance of developing breast cancer after using the Breast Health Decisions tool**
   - Strongly agree
   - Agree
   - Neutral
   - Disagree
   - Strongly disagree

2. **The Breast Health Decisions tool eased my worries and anxiety about my breast cancer risk**
   - Strongly agree
   - Agree
   - Neutral
   - Disagree
   - Strongly disagree

3. **I want to reduce my chance of developing breast cancer**
   - Strongly agree
   - Agree
   - Neutral
   - Disagree
   - Strongly disagree

4. **I am *__doing__* the following to reduce my chance of developing breast cancer [Please check all that apply]**
   - ☐ Taking medication that reduces my risk
   - ☐ Decreasing alcohol intake
   - ☐ Increasing exercise
   - ☐ Losing weight
   - ☐ Other __________ [fill in blank]
   - ☐ Nothing at this time

5. **I am *__considering__* the following to reduce my chance of developing breast cancer [Please check all that apply]**
   - ☐ Taking medication that reduces my risk
   - ☐ Decreasing alcohol intake
   - ☐ Increasing exercise
   - ☐ Losing weight
   - ☐ Other __________ [fill in blank]
   - ☐ Nothing at this time

If answering any of the questions in the study caused anxiety, we encourage you to send a message to wisdom@ucsf.edu or your breast health specialist if you have had your consultation.

***Message after survey completed:**
Thanks! You have completed your survey. We will check back in 3 months and ask you what decisions, if any, you have made due to information from the Breast Health Decisions Tool.

__**3-month follow up survey**__

1. **During the past 3 months, I have *__thought about__* my chances of developing breast cancer**
   a. Not at all
   b. Sometimes
   c. Often
   d. Almost all of the time

2. **During the past 3 months, I have *__worried about__* my chances of developing breast cancer**
   a. Not at all
   b. Sometimes
   c. Often
   d. Almost all of the time

3. **I want to reduce my chance of developing breast cancer**
   - Strongly agree
   - Agree
   - Neutral
   - Disagree
   - Strongly disagree

4. **I have discussed how to reduce my chance of developing breast cancer with my healthcare provider** YES/NO

   If YES →

   My healthcare provider believes I should reduce my chance of developing breast cancer
   - Strongly agree
   - Agreed
   - Neutral
   - Disagreed
   - Strongly disagree

   My healthcare provider recommended the following to reduce my chance of developing breast cancer [Please check all that apply]
   - ☐ Risk reducing medication (or chemoprevention)
   - ☐ Decreasing alcohol intake
   - ☐ Increasing exercise
   - ☐ Losing weight
   - ☐ Other __________ [fill in blank]
   - ☐ Nothing at this time

   If NO →

   I did not discuss how to reduce my chance of developing breast cancer with my healthcare provider because… [Please check all that apply]
   - ☐ I do not need to reduce my chance
   - ☐ I am already taking steps to reduce my chance
   - ☐ Personal motivation
   - ☐ Financial barriers
   - ☐ Access to health provider
   - ☐ Time limitations
   - ☐ Other _________

   I will discuss how to reduce my chance of developing breast cancer with my healthcare provider in the next 6 months
   - Strongly agree
   - Agree
   - Neutral

- Disagree
- Strongly disagree

5. **I am using risk reducing medication (i.e.: chemoprevention)** YES/NO

   If YES →

   The Breast Health Decisions Tool influenced my decision to use risk reducing medication
   - Strongly agree
   - Agree
   - Neutral
   - Disagree
   - Strongly disagree

   My healthcare provider influenced my decision to use risk reducing medication
   - Strongly agree
   - Agree
   - Neutral
   - Disagree
   - Strongly disagree
   - Not applicable

   If NO →

   I am not using risk reducing medication because … [Please check all that apply]
   - ☐ I do not need to reduce my chance
   - ☐ I am taking other steps to reduce my chance
   - ☐ Financial barrier
   - ☐ Personal motivation
   - ☐ Access to health provider
   - ☐ Time limitations
   - ☐ Fear of side effects
   - ☐ Other ___

6. **I am improving my diet to reduce my chance of developing breast cancer** YES/NO

   If YES →

   The Breast Health Decisions Tool influenced my decision improve my diet
   - Strongly agree
   - Agree
   - Neutral
   - Disagree
   - Strongly disagree

My healthcare provider influenced my decision to improve my diet

- Strongly agree
- Agree
- Neutral
- Disagree
- Strongly disagree
- Not applicable

If NO →

I am not improving my diet because … [Please check all that apply]

- ☐ I do not need to reduce my chance
- ☐ I am taking other steps to reduce my chances
- ☐ I already have a healthy diet
- ☐ Financial barrier
- ☐ Access to nutritionist
- ☐ Personal motivation
- ☐ Time limitations
- ☐ Other ___

7. **I am exercising more to reduce my chance of developing breast cancer** YES/NO

If YES →

The Breast Health Decisions Tool influenced my decision to exercise more

- Strongly agree
- Agree
- Neutral
- Disagree
- Strongly disagree

My healthcare provider influenced my decision to exercise more

- Strongly agree
- Agree
- Neutral
- Disagree
- Strongly disagree
- Not applicable

If NO →

I am not exercising more because … [Please check all that apply]

- ☐ I do not need to reduce my chance
- ☐ I am taking other steps to reduce my chance
- ☐ I already exercise
- ☐ Financial barrier
- ☐ Access to personal trainer

- ☐ Personal motivation
- ☐ Time limitations
- ☐ Other ____

8. **I am reducing my alcohol intake to reduce my chance of developing breast cancer** YES/NO

   If YES →

   The Breast Health Decisions Tool influenced my decision to reduce my alcohol intake
   - Strongly agree
   - Agree
   - Neutral
   - Disagree
   - Strongly disagree

   My healthcare provider influenced my decision to reduce my alcohol intake
   - Strongly agree
   - Agree
   - Neutral
   - Disagree
   - Strongly disagree
   - Not applicable

   If NO →

   I am not reducing my alcohol intake because … [Please check all that apply]
   - ☐ I do not need to reduce my chance
   - ☐ I am taking other steps to reduce my chance
   - ☐ I already minimize my alcohol intake
   - ☐ Financial barrier
   - ☐ Personal motivation
   - ☐ Time limitations
   - ☐ Other ____

9. **Would you like <u>support services</u> to help you reduce your chance of developing breast cancer?** YES/NO

If answering any of the questions in the study caused anxiety, we encourage you to send a message to wisdom@ucsf.edu or your breast health specialist if you have had your consultation.

Development and Pilot of an Online, Personalized Risk Assessment Tool for a Breast Cancer Precision Medicine Trial – Wang et al.

Instructions on Accessing the WISDOM Study Risk Assessment Tool: For your convenience and review, we have created a sample participant in our testing interface. Please follow these steps:

1. Visit: https://d1a000000iprkeao--uat.sandbox.my.site.com/wisdom/WSDlogin
2. Authenticate with the following information:
    a. ***High Risk Example Participant***
        i. Username: leah.uat@uat.com
        ii. Password: Leah.w1sd0mstudy
    b. ***Low Risk Example Participant***
        i. Username: llowrisk@athena.wsd8837
        ii. Password: Leah.w1sd0mstudy
3. If prompted to complete well-being questionnaire, click “Return to Study Portal”
4. Click “NEW: Breast Health Decisions Tool” in left blue column
