# Supplementary material for: Validation study of risk-reduction activities after personalized breast cancer education tool in the WISDOM study
Source: NPJ Breast Cancer. 2024 Oct 14;10:90. doi: 10.1038/s41523-024-00681-z (PMC11471852; doi:10.1038/s41523-024-00681-z)
Supplement: Supplementary file 2 — Reporting summary [file 41523_2024_681_MOESM2_ESM.pdf]

nature portfolio

Corresponding author(s): Laura Esserman, MD MBA (NPJBCANCER-02569R)

Last updated by author(s): 2/22/2024

Reporting Summary

Nature Portfolio wishes to improve the reproducibility of the work that we publish. This form provides structure for consistency and transparency in reporting. For further information on Nature Portfolio policies, see our Editorial Policies and the Editorial Policy Checklist.

Statistics

For all statistical analyses, confirm that the following items are present in the figure legend, table legend, main text, or Methods section.

n/a	Confirmed	
☐	☒	The exact sample size (*n*) for each experimental group/condition, given as a discrete number and unit of measurement
☒	☐	A statement on whether measurements were taken from distinct samples or whether the same sample was measured repeatedly
☒	☐	The statistical test(s) used AND whether they are one- or two-sided<br>*Only common tests should be described solely by name; describe more complex techniques in the Methods section.*
☒	☐	A description of all covariates tested
☒	☐	A description of any assumptions or corrections, such as tests of normality and adjustment for multiple comparisons
☒	☐	A full description of the statistical parameters including central tendency (e.g. means) or other basic estimates (e.g. regression coefficient) AND variation (e.g. standard deviation) or associated estimates of uncertainty (e.g. confidence intervals)
☐	☒	For null hypothesis testing, the test statistic (e.g. *F*, *t*, *r*) with confidence intervals, effect sizes, degrees of freedom and *P* value noted<br>*Give P values as exact values whenever suitable.*
☒	☐	For Bayesian analysis, information on the choice of priors and Markov chain Monte Carlo settings
☒	☐	For hierarchical and complex designs, identification of the appropriate level for tests and full reporting of outcomes
☒	☐	Estimates of effect sizes (e.g. Cohen's *d*, Pearson's *r*), indicating how they were calculated

Our web collection on statistics for biologists contains articles on many of the points above.

Software and code

Policy information about availability of computer code

Data collection	R studio version 1.0.153
Data analysis	R studio version 1.0.153

For manuscripts utilizing custom algorithms or software that are central to the research but not yet described in published literature, software must be made available to editors and reviewers. We strongly encourage code deposition in a community repository (e.g. GitHub). See the Nature Portfolio guidelines for submitting code & software for further information.

Data

Policy information about availability of data

All manuscripts must include a data availability statement. This statement should provide the following information, where applicable:

- Accession codes, unique identifiers, or web links for publicly available datasets
- A description of any restrictions on data availability
- For clinical datasets or third party data, please ensure that the statement adheres to our policy

The datasets used and analyzed for the current study are available on Open Science Framework and can be accessed from the following link: http://dx.doi.org/10.17605/OSF.IO/YJ782

Research involving human participants, their data, or biological material

Policy information about studies with human participants or human data. See also policy information about sex, gender (identity/presentation), and sexual orientation and race, ethnicity and racism.

Reporting on sex and gender	All participants were of female, and therefore findings are only applicable to the female sex.
Reporting on race, ethnicity, or other socially relevant groupings	N/A
Population characteristics	N/A
Recruitment	Participants of the WISDOM Study were recruited digitally through outreach, in-person flyers, online social media groups. There is possible selection bias, as it can self-select for individuals motivated to better understand their own health and contribute to the scientific community, and likely those who have higher educational background and reliable internet access. For the Breast Health Decisions tool survey recruitment, eligible WISDOM participants were contacted through email and/or phone by their breast health specialist. And they also had the opportunity to go through the tool by themselves. Again, this could pose selection bias with certain participant characteristics perhaps predisposing them to utilize the tool.
Ethics oversight	University of California, San Francisco Institutional Review Board

Note that full information on the approval of the study protocol must also be provided in the manuscript.

Field-specific reporting

Please select the one below that is the best fit for your research. If you are not sure, read the appropriate sections before making your selection.

☒ Life sciences ☐ Behavioural & social sciences ☐ Ecological, evolutionary & environmental sciences

For a reference copy of the document with all sections, see nature.com/documents/nr-reporting-summary-flat.pdf

Life sciences study design

All studies must disclose on these points even when the disclosure is negative.

Sample size	We planned for at least 100 participants for our validation, without predetermined sample size selection calculations. We chose this sample size because it is the typical minimum sample size to use for a large population. We were able to successfully obtain more than 300 participant responses to our feedback survey, which greatly exceeded our 100 participant goal. We believed this was sufficient for the nature of our research, which involved primarily descriptive analysis and did not include regression modeling.
Data exclusions	We excluded two participants with "stop screening" or "start screening at age 50" recommendations since this meant that their breast cancer risk profile no longer fit our high risk category. We also excluded 13 participants who completed their survey after being designated low risk, a fact that we could only look at retrospectively after data collection.
Replication	N/A
Randomization	This is not relevant to the specific survey collection data we performed. The purpose of our study was to validate our online Breast Health Decisions tool, which involved a pre-post study design and therefore did not utilize randomization.
Blinding	Blinding was not relevant to the study since we used a pre-post study design and did not perform specific allocations or randomization.

Reporting for specific materials, systems and methods

We require information from authors about some types of materials, experimental systems and methods used in many studies. Here, indicate whether each material, system or method listed is relevant to your study. If you are not sure if a list item applies to your research, read the appropriate section before selecting a response.

Materials & experimental systems

n/a	Involved in the study
☒	☐ Antibodies
☒	☐ Eukaryotic cell lines
☒	☐ Palaeontology and archaeology
☒	☐ Animals and other organisms
☐	☒ Clinical data
☒	☐ Dual use research of concern
☒	☐ Plants

Methods

n/a	Involved in the study
☒	☐ ChIP-seq
☒	☐ Flow cytometry
☒	☐ MRI-based neuroimaging

Clinical data

Policy information about clinical studies

All manuscripts should comply with the ICMJE guidelines for publication of clinical research and a completed CONSORT checklist must be included with all submissions.

Clinical trial registration	NCT02620852
Study protocol	WISDOM Study Trial protocol is available on clinicaltrials.gov
Data collection	All data was collected electronically through a secure Salesforce platform which hosted participants' WISDOM portals. Recruitment began on August 31, 2016. Primary completion date for collection is December 1, 2024. Study completion date is March 1, 2025.
Outcomes	The results from our validation study of the Breast Health Decisions tool is only one subset of the WISDOM Study's secondary end-points: whether understanding personalized risk results in uptake of breast cancer prevention strategies
